# Gestalt Reasoning with Conjunctions and Disjunctions

**DOI:** 10.1371/journal.pone.0151774

**Published:** 2016-03-17

**Authors:** Magda L. Dumitru, Gitte H. Joergensen

**Affiliations:** 1 Department of Cognitive Science, Macquarie University, Sydney, NSW, Australia; 2 Department of Cognitive Science, METU, Ankara, Turkey; 3 Department of Psychology, University of York, Heslington, York, United Kingdom; 4 Department of Psychology, University of Connecticut, Storrs, CT, United States of America; Cardiff University, UNITED KINGDOM

## Abstract

Reasoning, solving mathematical equations, or planning written and spoken sentences all must factor in stimuli perceptual properties. Indeed, thinking processes are inspired by and subsequently fitted to concrete objects and situations. It is therefore reasonable to expect that the mental representations evoked when people solve these seemingly abstract tasks should interact with the properties of the manipulated stimuli. Here, we investigated the mental representations evoked by conjunction and disjunction expressions in language-picture matching tasks. We hypothesised that, if these representations have been derived using key Gestalt principles, reasoners should use perceptual compatibility to gauge the goodness of fit between conjunction/disjunction descriptions (e.g., *the purple* ***and/ or*** *the green*) and corresponding binary visual displays. Indeed, the results of three experimental studies demonstrate that reasoners associate conjunction descriptions with perceptually-dependent stimuli and disjunction descriptions with perceptually-independent stimuli, where visual dependency status follows the key Gestalt principles of common fate, proximity, and similarity.

## Introduction

People have many options available for solving complex tasks in real-life situations. One option is to apply the rules of propositional logic [1–4] and identify the relations (e.g., cause, consequence, condition, coordination) holding between the main action to be performed and further associated actions. For example, a medical emergency protocol recommends US service providers that “If no shock is indicated, consider termination of resuscitation protocol or transport patient immediately”. To evaluate this statement, people need to combine the truth-values of the coordination statement (composed of two clauses linked by the disjunction connective ‘or’) and of the conditional statement (introduced by the connective ‘if’). Specifically, disjunction statements are true unless both parts fail to obtain (i.e., resuscitation continues and patient is not transported) and conditional statements are true unless the first part obtains while the second part does not (i.e., there is no shock indicated, yet resuscitation continues and patient is not transported). Nevertheless, experimental evidence increasingly suggests that naive reasoners forego formal rules and instead draw on general cognitive mechanisms when processing complex information. Accordingly, their responses are often modulated by stimuli perceptual properties [5], by metaphors that ground incoming information onto basic action patterns [6–7], or by mental models that represent, in analogical form, key elements in a message and the relationships between them [8–10].

The present paper builds on previous findings [5] suggesting that a general mechanism of information processing, namely parsing (aka ‘chunking’) shapes reasoning with conjunctions and disjunctions. While there is ample experimental evidence that incoming information is often chunked into smaller units to facilitate processing [11–12] the nature and properties of these chunks are largely unknown. Here, we advance the hypothesis that, when processing conjunctions and disjunctions, reasoners instantly build Gestalt-like representations (one and two mental objects respectively—cf. [5]), which generate perceptual compatibility effects predicted by key Gestalt principles (i.e., common fate, proximity, and similarity). As such, Gestalt representations of conjunctions and disjunctions are not unlike concrete object representations stored in long-term memory and therefore they should interact with the properties of the perceptual stimuli that reasoners manipulate when solving a particular task. The remainder of this paper is organised as follows. After summarising the main tenets of Gestalt theory, we outline the advantages of the earliest analogy-based approaches, in particular the important progress made by model theory towards achieving a psychologically plausible account of conjunction and disjunction representations. Subsequently, we present three experimental studies investigating a novel account according to which reasoners build Gestalt-like conjunction and disjunction representations in online language-picture matching tasks. We expect that, unlike previous reasoning accounts, Gestalt reasoning based on indirect analogies can successfully predict the observed response patterns.

Gestalt psychologists ever since Koehler [13], Koffka [14], and Wertheimer [15] have developed a phenomenological appraisal of perceptual processes according to which “the whole is different from the sum of its parts”. In their view, perceptual information must go well beyond stimuli properties or bodily sensations for people to be able to group them into meaningful units, which facilitate mental processes and attention allocation. Moreover, evidence from several cognitive domains (for reviews see Tversky [16–17]) demonstrates that grouping is a general process of the mind, hence is not limited to perception. Studies of cognitive maps, for instance, have revealed that grouping can readily occur in memory, as individuals were faster to determine which of a pair of cities was farther east or farther north when the cities were located in different states or countries than when they were in the same state or country [18–19]. Likewise, people tend to perceive members of the same social or political group as more similar than members from different groups even when asked to judge them on irrelevant attributes [20].

Several principles have been described for organising stimuli into groups, of which the most important is the principle of common fate. Simply put, adults as well as children [21] will tend to group together elements that move together. For instance, they will easily target a flock of birds flying in the same direction. Grouping may also depend on other types of perceptual information when stimuli are not moving, namely on whether they are close to each other or similar to each other (e.g., in shape, color, or size). In other words, grouping can also happen through the Gestalt principles of proximity and similarity. We hypothesize that individuals also observe key Gestalt principles when reasoning with conjunctions (e.g., *the purple and the green*) and disjunctions (e.g., *the purple or the green*) by drawing on their experience with previous conjunction and disjunction situations. We thereby use insights from embodied and grounded cognition theories [22–26] according to which stimuli properties play a key role in simulation retrieval. To wit, coordination simulations are grounded in individuals’ experience with selecting both items mentioned in conjunction contexts (e.g., *Have a cup of coffee and a biscuit*!*)* and with selecting one of two items mentioned in disjunction contexts (e.g., *Have a biscuit or a fruit*!*)*. Differently put, conjunctions and disjunctions evoke one-Gestalt simulations and two-Gestalts simulations respectively.

Embodied and grounded cognition theories further predict more efficient processing for descriptions evoking simulations that are compatible with corresponding perceptual stimuli. As shown in Dumitru [5], the two items in the simulation of conjunction descriptions (e.g., *the purple and the green*) form a single conceptual unit, which makes conjunction simulation compatible with jointly-moving (i.e., perceptually dependent) stimuli. Also, the two items in the simulation of disjunction descriptions (e.g., *the purple or the green*) were shown to form two conceptual units, which makes disjunction simulation incompatible with jointly-moving stimuli. Further, simulations of any kind are only partly compatible with ambiguous stimuli (e.g., stationary visual displays), as they provide insufficient information for assessing dependency status. Accordingly, Dumitru [5] reported high validation scores for compatible stimuli, low validation scores for incompatible stimuli, and mitigated scores for ambiguous stimuli.

The reliance of Gestalt reasoning on real-life situations makes it compatible with mental-model theories [8–10], which hold that reasoners construct simplified representations (i.e., “models”) of concrete situations by assigning each referent in real life an analogous token in a mental model and each relation between referents an analogous relation between tokens in the same model. So, for example, we might draw the conclusion that “Matthew is to the right of John” from the premises “Matthew is to the right of Mark” and “Mark is to the right of John” if we pictured a situation where the three people sit at a rectangular table as in Leonardo Da Vinci’s *The Last Supper*, but not at a triangular, round, or very small table. Similarly, mental models theory holds that reasoning with conjunctions involves building a single model, whereas reasoning with disjunctions involves building no less than three models [10]. In particular, the assertion *There is a circle and a triangle* evokes a situation where both a circle and a triangle are present, whereas the assertion *There is a circle or a triangle* evokes a situation where only a circle is present, another situation where only a triangle is present, and—if one interprets disjunction inclusively—yet another situation where both a circle and a triangle are present. By having disjunction optionally represent two or three situations, model theory is able to account for people’s spontaneous tendency to interpret disjunction either exclusively or inclusively that is, for either invalidating or validating disjunctions describing two objects. By positing two default disjunction representations (inclusive and exclusive) and a costly mechanism for updating the number of models, the theory reconciles previous contradictory findings in the disjunction representation literature [27–29]. However, the theory cannot obviate alternative accounts of model updating [30].

The big strides made by model theory towards providing a psychologically plausible account of reasoning with conjunctions and disjunctions are largely due to the analogical mechanisms they posit for capturing reasoners’ mental processes in real time. Nevertheless, the results reported in Dumitru [5], which are easily explained by Gestalt-based approaches, cannot be accounted for by model theory. This is likely to be due to what we consider to be a crucial difference in the theoretical status of mental representations that each theory has assumed. In particular, mental models theory posits direct analogies between tokens and text referents and therefore mental transformations cannot modify the relationships between them. As such, models are merely static snapshots of possible situations. In contrast, Gestalt reasoning posits only indirectly analogies between Gestalts and text referents, as the former result from a dynamic reasoning process inherent to task-solving mechanisms, which alters the relations between tokens. As a result, structural analogies between concrete visual stimuli and object-evoking names in conjunction and disjunction descriptions are no longer direct; the two tokens corresponding to distinct real-life objects are merged into one and two Gestalts respectively. Therefore we might expect reasoners to validate “the purple and the green”, for example, as a description of a display containing a purple object and a green object at higher rates when the perceptual properties of the two objects are such that the objects can be merged into a single mental representation that is, when they are likely to form a single Gestalt. Similarly, we might expect reasoners to validate “the purple or the green” as a description of a display containing two objects, one purple and the other green, at higher rates when the perceptual properties of the two objects are such that the objects cannot be merged into a single mental representation but must be considered as being independent that is, when they are likely to form two Gestalts.

Indeed, research on mathematical reasoning suggests that, when performing calculations or solving algebraic equations, reasoners need to move and transform mathematical symbols in their mind [31–33]. For example mathematical algorithms have been shown to be grounded in the visual format of notational displays (e.g., physical spacing or variable names) such that manipulating the visual grouping of elements in an equation impacts the order in which algebraic operations are performed ([34]). Research on sentence structure production has also documented perceptual grouping effects. As reported by Myachykov and Tomlin [35], visual primes determine the choice of sentence structure. In particular, concurrent visual cues were shown to nudge speakers towards selecting either the active-voice sentence *The shark ate the fish* or the passive-voice sentence *The fish was eaten by a shark* as an appropriate description (also see [36–37]). Indeed, subsequent research by Scheepers et al. [38] highlighted a common basis of structural representations in language and in mathematics by showing that notational displays of algebraic equations directly influence sentence comprehension.

An important consequence of operating with indirect analogies in Gestalt-reasoning is that reasoners might not access these representations consciously, hence may not be able to justify their preference for validating or invalidating conjunction and disjunction descriptions in specific perceptual contexts. In contrast, model-theoretical approaches predict that reasoners should be able to report on the number of possible situations they are imagining. Recent evidence that Gestalt processing takes places at the sub-conscious level comes from an eye-movements study [39] showing that, when participants are presented with two stationary pictures (e.g., the picture of an ant next to the picture of a cloud), they shift their gaze faster from the picture representing the first word to the picture representing the second word while hearing conjunction descriptions (e.g., *There is an ant and a cloud*) than when hearing disjunction descriptions (e.g., *There is an ant or a cloud*). Indeed, difficulties in shifting attention between objects (here, between two Gestalts in disjunction trials) but not between object parts (here, between the two halves of a single Gestalt in conjunction trials) are well documented in the object-processing literature [40–41].

In summary, we predict that, when perceptual stimuli are compatible with Gestalt conjunction and disjunction representations, they should generate higher validation scores and faster processing of their corresponding conjunction and disjunction descriptions, whereas stimuli that are perceptually incompatible with Gestalt representations should generate lower validation scores and slower processing of the same descriptions. The evidence available so far in support of Gestalt reasoning [5] allows us to infer that jointly-moving stimuli are exclusively compatible with single-Gestalt simulations, given that joint motion is a robust cue to spatiotemporal dependency [42–46]. Therefore jointly-moving stimuli are good matches for conjunction descriptions and bad matches for disjunction descriptions. This explains why they yielded high validation scores for the former and low validation scores for the latter. However, these findings only go halfway towards demonstrating that coordination simulations are Gestalt-like. We also need to determine whether the reverse is true, namely that independently-moving stimuli are bad matches for conjunction descriptions and good matches for disjunction descriptions. Moreover, we also need to determine whether coordination representations are susceptible equally well to perceptual compatibility effects represented by (in) dependent motion, thus illustrating the Gestalt principle of common fate, and to perceptual compatibility effects represented by similarity and by proximity, which illustrate the Gestalt principles of similarity and proximity respectively. The three experimental studies we present here will determine which perceptual properties visual stimuli should have for reasoners to validate them as good matches for conjunction descriptions and for disjunction descriptions. In particular, we aimed to show that individuals apply the Gestalt principles of common fate, proximity, and similarity when matching binary visual displays to conjunction descriptions (e.g., *the purple and the green*) and to disjunction descriptions (e.g., *the purple or the green*).

For each trial, we recorded accuracy scores (responses were coded as ‘1’ if participants validated the conjunction/disjunction description in a particular trial, or as ‘0’ if they failed to validate the description) as well as response times (RTs measured in ms). While we were mostly interested in establishing a link between overt accuracy scores and stimuli perceptual properties, we also expected differences in RTs when participants processed one versus two Gestalts, based on well-established findings from the visual perception literature that humans process global patterns faster than local patterns. In particular, Gestalt-like processes sensitive to global image configurations have been shown to systematically dominate local feature processing in human pattern perception (i.e., the ‘global precedence’ principle–[47–49]) such that visual displays containing a single Gestalt are processed faster than visual displays containing several Gestalts. We were thus expecting the meaning of the connective “and” versus “or” to modulate any global precedence effects we might observe, such that these effects should be weakened if not reversed for the latter compared to the former. Response-times data can thus represent a particularly useful tool for eliciting participants’ reaction to matches and to mismatches between visual displays on the one hand, and the meaning of conjunction and disjunction descriptions on the other hand. In other words, response times provide information on covert reasoning mechanisms that accuracy data alone cannot supply.

## Experiment 1: Common Fate

Experiment 1 investigated the effects of common fate on conjunction and disjunction processing in terms of accuracy scores and response times. The Gestalt principle of common fate allows us to predict that, when two stimuli are removed and replaced simultaneously, participants perceive them as forming a single Gestalt. As a result, they should validate these simultaneous dynamic displays as successful matches of conjunction descriptions but not of disjunction descriptions. Conversely, the principle of common fate allows us to predict that, when stimuli are removed and replaced alternatively (i.e., one by one), participants perceive them as forming two separate Gestalts. As a result, they should validate these alternative dynamic displays as successful matches of disjunction descriptions but not of conjunction descriptions. In terms of RT values, global precedence principles should facilitate processing of one-Gestalt displays, where stimuli are removed simultaneously, but not of two-Gestalts displays, where stimuli are removed alternatively. Nevertheless, we expected slower processing of one-Gestalt displays and faster processing of two-Gestalts displays in disjunction trials than in conjunction trials, resulting from an interaction between global-precedence principles and Gestalt-reasoning principles, as explained above.

### Method

#### Ethics statement

The experiments reported here were completed in accordance with the Declaration of Helsinki and also followed the ethics guidelines set forth by Macquarie University Human Research Ethics Committee, which approved this study with the reference number HE28SEP2007-D05413. Participants were informed that their data would be treated confidentially and that they could terminate the experiment at any time without citing any reason. All study participants signed an informed consent from before taking part, describing the potential risks and benefits of participation. Upon agreeing to these conditions, participants were provided with additional instructions and the experimental materials. Data from the three studies reported here are available at https://osf.io/4g62y/.

#### Participants

Twenty native speakers of English volunteered to participate in individual sessions lasting about 20 minutes in return for course credit. They all reported normal or corrected-to-normal vision.

#### Stimuli

**Fig 1:**
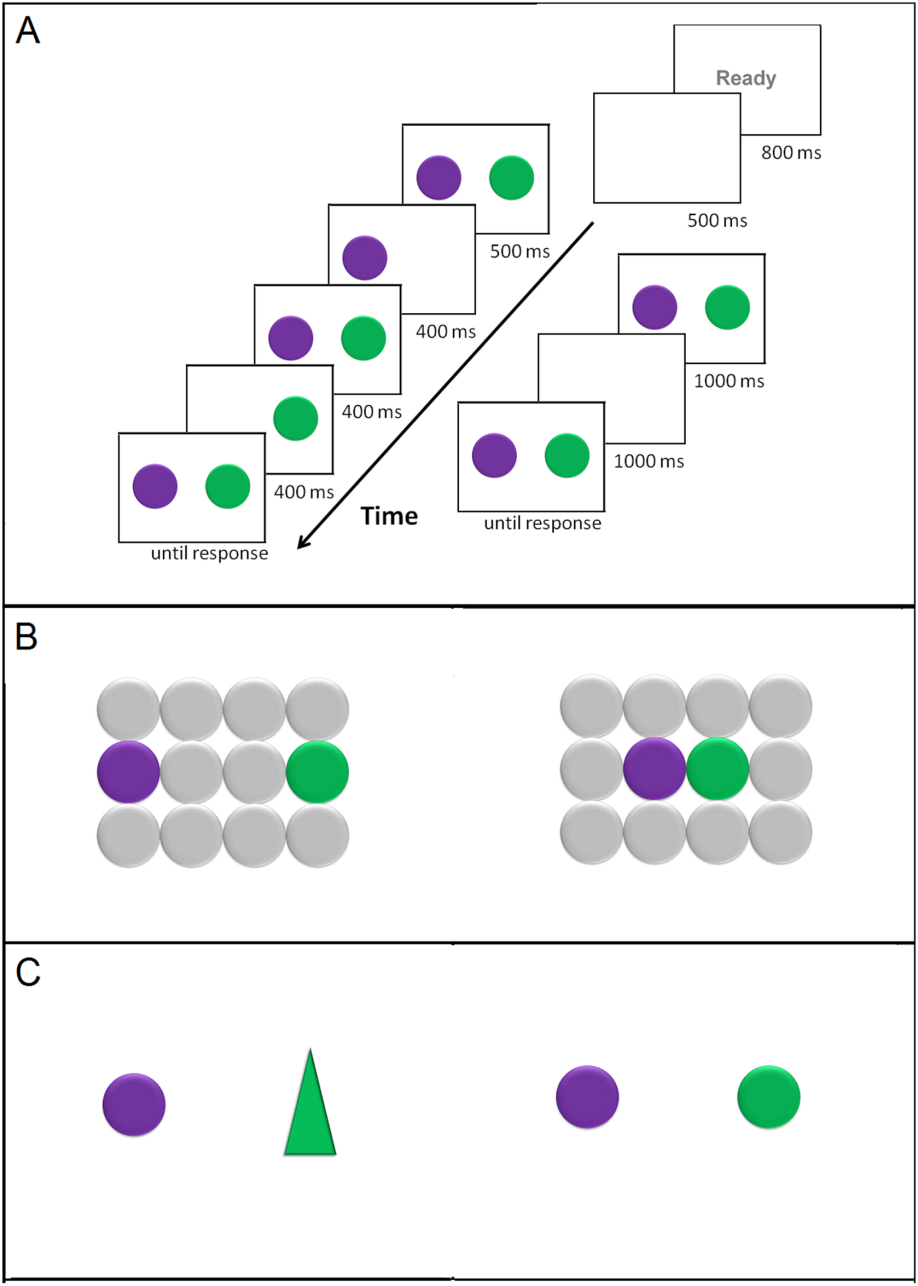
Example of a sequence of events on a typical trial for each condition in each experiment. Experiment 1 (A) investigated the Gestalt principle of common fate: Disks were either removed alternatively (left side panels) or simultaneously (right side panels). Experiment 2 (B) investigated the Gestalt principle of proximity: Disks were either placed far apart (left side panel) or close together (right side panel). Experiment 3 (C) investigated the Gestalt principle of similarity: Figures were either of the same shape (left panel) or of different shapes (right panel). All panels featuring visual stimuli were accompanied by a matching description written in the superior quarter of the screen, which contained a conjunction word (*the purple* ***and*** *the green*) or a disjunction word (*the purple* ***or*** *the green*).

Stimuli in the experimental condition of interest (where both visual stimuli matched the words mentioned in conjunction and disjunction descriptions) consisted of 24 visual displays. Each display contained two colored disks as seen in Fig 1A, and was accompanied by a conjunction description (e.g., *the purple* ***and*** *the green*) or by a disjunction description (e.g., *the purple* ***or*** *the green*). In half of the trials, the disks were removed alternatively such that at least one disk was displayed at all times. In the other half, disks were removed and replaced simultaneously such that both disks were either present or absent at all times. We also included 72 filler trials illustrating the remaining possibilities, namely situations where only one of the disks (i.e., the right or the left) matching the colors mentioned in the coordination description was removed (i.e., situations incompatible with conjunction simulations), and situations where both disks being removed mismatched the descriptions (i.e., situations incompatible with both conjunction and disjunction simulations).

#### Design and procedure

The experiment followed a 2 (Coordination: AND vs. OR) x 2 (Visual Stimuli: Two-Gestalts vs. One-Gestalt) factorial design. For filler trials, the two coordination conditions were associated only with alternative removal, as a single stimulus can be removed in only one fashion. We were thus exclusively interested in participants’ performance in conditions where both stimuli were removed, whether simultaneously or alternatively. Participants were informed that they should carefully watch the disks and that, once the disks have stopped moving, they should press the right button of a response box if they believed the dynamic display matched the descriptions, and the left button otherwise (counterbalanced). As became obvious during the practice session, early response attempts were ineffective for concluding a trial. Participants were further informed that, in filler trials, single removal was compatible with disjunction but not with conjunction descriptions and that removal of two disks of non-matching colors was always incompatible with coordination descriptions. However, participants were not informed as to the ‘correct answer’ in the conditions of interest (where disks’ colors matched both colors mentioned in the descriptions). Hence their decisions reflected spontaneous preferences, as discussed in previous reasoning studies (e.g., [30, 5]).

The structure of a trial was as follows. First, the word “Ready” appeared in the centre of the screen for 1000 ms, followed by a 500 ms blank screen, then by a binary visual display. In the alternative condition, the display remained visible for 500 ms and was followed by successive 400 ms displays featuring the left disk, both disks, the right disks, and both disks again. The final frame remained visible until response. In the simultaneous condition, the display remained visible for 1000 ms and was followed by a 1000 ms blank display, and then by a binary display. The final frame remained visible until response. The experiment consisted of 6 practice trials and 96 experimental trials (24 target trials and 72 fillers) presented in individually randomized orders in two equal blocks. The dependent variables were the percentage of ‘yes’ responses and the response times across trials.

### Results and discussion

**Fig 2:**
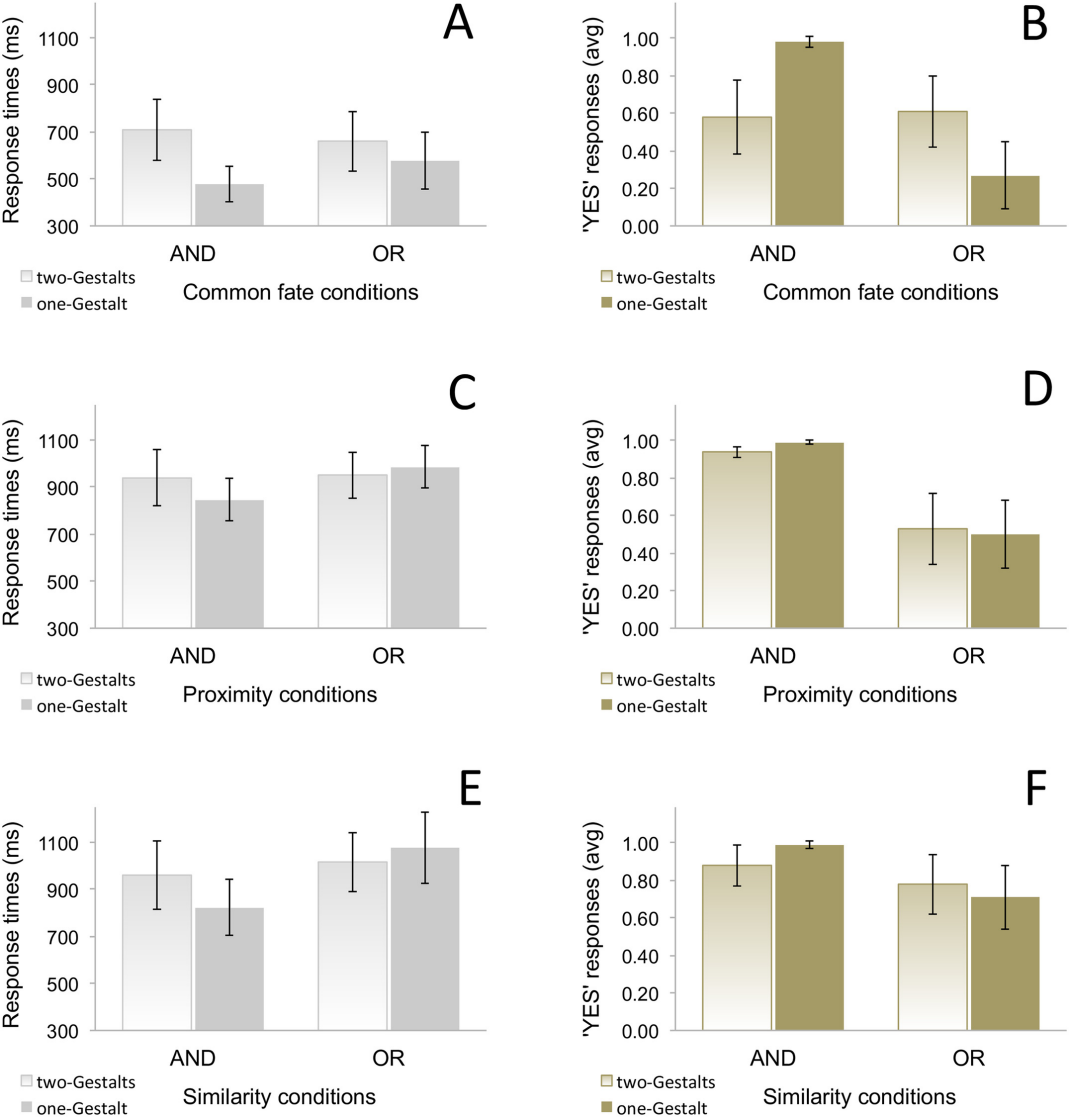
Average response times and average ‘yes’ responses across conditions in Experiment 1 (A & B), in Experiment 2 (C & D), and in Experiment 3 (E & F). Error bars represent 95% confidence intervals. Response times were lowest and accuracy scores highest for conjunctions in one-Gestalt conditions. Conversely, response times were lowest and accuracy scores highest for disjunctions in two-Gestalts conditions.

Fig 2B summarizes validation patterns (i.e., average proportion of ‘yes’ responses) across trials. The accuracy scores were analysed using logit mixed-effect models ([50]) in the R statistical language ([51]). The first model included the binomial dependent variable ‘response accuracy’ and ‘participants’ as random factors, as well as the predictor variables ‘coordination’ and ‘visual stimuli’ as fixed factors. We contrasted this model with a model that further included an interaction between the fixed factors. Model fit was assessed using chi-square tests on the log-likelihood values. We found the second model to be a better fit for the data, χ2 = 108.02, p < .001. In particular, the log odds of validating disjunctions decreased by 5.8 (Std. Error = .819, p < .001) compared to conjunctions in trials where stimuli formed one Gestalt (i.e., they appeared simultaneously). Results for filler trials were well above chance, suggesting that participants could retrieve the meaning of conjunctions and disjunctions, as instructed prior to the experimental session. For conjunction first, second, and no-match trials, validation scores averaged .97, .98, and .99 respectively. For disjunction first, second, and no-match trials, validation scores averaged .94, .96, and .95 respectively.

Fig 2A summarizes the average response times across trials. RT scores were analysed using linear mixed-effects regression, LMER, with the lme4 package in R ([52]). We compared two models, with and without an interaction between the fixed factors ‘coordination’ and ‘visual stimuli’ and found the interaction model to be a better fit, as assessed using chi-square tests on maximum likelihood values, χ2 = 5.59, p = .018. Participants responded on average 160.07 ms later (Std. Error = 67.46, t = 2.37) in one-Gestalt trials (where stimuli appeared simultaneously) than in two-Gestalts trials (where stimuli appeared alternatively) for the disjunction condition. These results confirm the validation patterns above, thus suggesting a preference for associating conjunction simulations with stimuli forming a single Gestalt and disjunction simulations with stimuli forming two Gestalts.

An important question arising at this point is whether our dynamic experimental setup might have affected language reference verification and hence RT values, especially that meaning and verification processes differ when understanding conjunctions and disjunctions. In particular, stimuli perceptual properties might have nudged participants to stop halfway during the meaning verification process of the two nouns mentioned in disjunction trials given that verifying the reference of the first noun already satisfies disjunction validation. Indeed, recent findings suggest that language comprehension and reference verification unfold incrementally (e.g., [52]) such that disjunction meaning directly affects language verification. However, experimental evidence also suggests that adults as well as children manifest an irrepressible need towards verifying the reference of all words in a sentence (for a review see [53], in press) such that, as soon as a noun refers to an object, they are more likely to inspect that particular object rather than any other semantically associated item in a visual display. We confirmed these findings for conjunction and for disjunction sentences in an eye-tracking study ([39]) where we reported that, in the condition of interest, where the two visual stimuli matched the two nouns mentioned, participants readily shifted their gaze towards the referent of the second noun in both conjunction and disjunction trials.

Moreover, evidence from the visual perception literature suggests that, if anything, dynamic displays intensify the reference verification process. In particular, abrupt visual stimuli onsets were shown to capture attention even when they were unrelated to the items that participants were searching for (e.g., [54]). Indeed, our data demonstrate that conjunctions and disjunctions were easy to understand and verify in dynamic displays, as we obtained fast responses across the board and low error rates in filler trials. As predicted by Gestalt reasoning, the ‘two-Gestalts’ conditions yielded high validation scores in disjunction trials and low validation scores in conjunction trials. Conversely, the ‘one-Gestalt’ condition yielded low validation scores in disjunction trials and high validation scores in conjunction trials. This pattern of results cannot be accounted for by other theoretical approaches such as linguistic pragmatics, where no relationship can be established between stimuli perceptual properties and validation scores. Moreover, even if we assumed that, in this theoretical framework, the assumption were valid that conjunction meaning only matched simultaneous binary displays, we could not explain why the same simultaneous displays were easy to process across the board, thus yielding ‘enriched’ disjunction interpretations. Specifically, if we adopted a linguistic pragmatics account, we would maintain that participants validated less disjunction trials in the ‘simultaneous’ than in the ‘alternative’ condition because the former context was easier and thus allowed them to switch from the default inclusive interpretation to the more relevant exclusive interpretation of disjunction meaning. Rather, the overall low RT values in our experiment suggest that participants found it particularly easy to retrieve and verify the meaning of conjunctions and disjunctions in all dynamic contexts.

## Experiment 2: Proximity

Experiment 2 investigated the effects of stimuli proximity on conjunction and disjunction processing in terms of accuracy scores and response times. Gestalt principles predict that participants perceive stimuli that are placed close to each other as forming a single Gestalt. Therefore they should validate such proximal displays as successful matches for conjunction descriptions but not for disjunction descriptions. Conversely, Gestalt principles predict that participants perceive stimuli placed far from each other as forming two separate Gestalts. Therefore they should validate such distal displays as successful matches of disjunction descriptions but not of conjunction descriptions. In terms of RT values, global precedence principles should facilitate processing of one-Gestalt displays, where stimuli are proximally placed, but not of two-Gestalts displays, where stimuli are distally placed. Nevertheless, Gestalt-reasoning principles might weaken or even reverse these effects, hence induce slower processing of one-Gestalt displays and faster processing of two-Gestalts displays in disjunction trials than in conjunction trials.

### Method

#### Participants

Twenty participants were selected, as in Experiment 1.

#### Stimuli

Stimuli in the experimental condition of interest consisted of 36 visual displays, each of them containing two disks of different colors placed in a grid of grey disks, as seen in Fig 1B. The two disks matched the colors mentioned in an accompanying conjunction or disjunction description and were placed far from each other in half of the trials and close to each other in the other half. The number of trials was calculated so as to exhaust all horizontal combinations for the two proximity conditions. We also included 72 filler trials, where displays contained a single disk whose color matched one of the colors mentioned in the description (i.e., situations incompatible with conjunction simulations), or a single disk whose color mismatched both colors mentioned in the coordination descriptions (i.e., situation incompatible with both conjunction and disjunction simulations).

#### Design and procedure

The experiment followed a 2 (Coordination: AND vs. OR) x 2 Visual Stimuli: Two-Gestalts vs. One-Gestalt) factorial design. For filler trials containing a single disk, we only contrasted the two connective conditions, as the concepts “close” and “far” cannot be defined with respect to a single stimulus. As in Experiment 1, participants were informed as to what the correct responses to filler trials should be. In particular, a visual display where one disk matched the first or the second color mentioned was compatible with disjunction but not with conjunction descriptions, whereas a visual display where the disk matched neither color mentioned was always incompatible with coordination descriptions. Participants were not given any further information about the correct answer in the conditions of interest, but were encouraged to follow their intuition.

On a typical trial, the word “Ready” first appeared in the centre of the screen for 800 ms, followed by a 500 ms blank screen and by a visual display that remained visible until response. The experiment consisted of 6 practice trials and of 108 experimental trials (36 target trials and 72 fillers) presented in individually randomized orders in two equal blocks. As in Experiment 1, the dependent variables were the percentage of ‘yes’ responses and the response times across trials.

### Results and discussion

Fig 2D summarizes the validation patterns (i.e., average proportion of ‘yes’ responses) across conditions. As in Experiment 1, we used logit analyses to calculate accuracy scores for two models. The first model included the binomial dependent variable ‘response accuracy’ and ‘participants’ as random factors, as well as the predictor variables ‘coordination’ and ‘visual stimuli’ as fixed factors. We contrasted this model with a model including an interaction between the fixed factors. The second model was a better fit for the data, χ2 = 3.56, p = .058. In particular, the log odds of validating disjunctions increased by 1.47 (Std. Error = 0.85, p = .008) compared to conjunction trials when stimuli formed one Gestalt (i.e., they were far apart). Results for filler trials in the single condition tested (i.e., where a single disk was visible, whose color mismatched one or both color names mentioned in the coordination descriptions) were well above chance. For conjunction first, second, and no-match trials, validation scores averaged .97, .92, and 1.00 respectively. For disjunction first, second, and no-match trials, validation scores averaged .93, .97, and .99 respectively.

Fig 2C summarizes the average response times across conditions. We used LMER to analyse RT scores across two models, one with and the other without an interaction between the fixed factors ‘coordination’ and ‘visual stimuli’. The interaction model was a better fit, χ2 = 5.18, p = .022. Participants responded on average 125.42 ms later (Std. Error = 54.99, t = 2.28) to one-Gestalt displays (where stimuli were proximally placed) than to two-Gestalts displays (where stimuli were distally placed) in disjunction trials. The results confirm the validation patterns above, thus suggesting a preference for associating conjunction simulations with proximally-displayed stimuli and disjunction simulations with distally-displayed stimuli.

However, we expected a stronger interaction between the fixed factors for both validation scores and RT values based on previous findings ([55–57]) that the principle of proximity operates relatively high in the hierarchy of Gestalt principles (e.g., prior to shape similarity). However, our data are very sensible in the light of supplementary evidence ([58–59]) that global precedence effects are weakened when visual stimuli are placed against a background formed of similar shapes. In such cases, target and background elements could no longer be clearly distinguished from each other. Indeed, according to Han, Humphreys, & Chen ([60–61]), a novel Gestalt principle (i.e., the principle of uniform connectedness) operates over regions of uniform visual properties (e.g., similar luminance, color, or texture) to organize them in a single perceptual unit. In our study, targets and background were composed of similar elements (equal-size disks), hence the uniform elements between targets must have functioned as reliable links between them. As a result, it was difficult to perceive the targets thus linked to each other as separate Gestalts when placed distally.

## Experiment 3: Similarity

Experiment 3 investigated the effects of stimuli similarity on conjunction and disjunction processing in terms of accuracy scores and response times. Based on Gestalt principles, we predicted that participants would perceive stimuli having the same shape (i.e., two disks) as forming a single Gestalt. Therefore they should validate such displays as successful matches of conjunction descriptions but not of disjunction descriptions. Conversely, we predicted that participants would perceive stimuli having different shapes (e.g., a disk and a triangle) as forming two separate Gestalts. Therefore they should validate such displays as successful matches of disjunction descriptions but not of conjunction descriptions. In terms of RT values, global precedence principles should facilitate processing of one-Gestalt displays, where stimuli are similar to each other but not of two-Gestalts displays, where stimuli are dissimilar from each other. As in the previous experiments, however, Gestalt-reasoning principles might slow down and even reverse the processing of one-Gestalt displays and instead facilitate the processing of two-Gestalts displays in disjunction trials compared to conjunction trials.

### Method

#### Participants

Twenty participants were selected, as in the previous two experiments.

#### Stimuli

Stimuli consisted of 24 visual displays containing two geometrical figures whose colors matched the colors mentioned in the accompanying conjunction or disjunction description, as seen in Fig 1C. Half of the trials included figures that were similar in form (i.e., two disks); the other half included figures that were dissimilar in form (i.e., a disk and a triangle). We also constructed 48 filler trials where figures were all similar (i.e., two disks) illustrating the remaining matching possibilities, namely binary displays where the color of one disk (i.e., the left or the right) matched one of the colors mentioned in the coordination description (i.e., situations incompatible with conjunction simulations), as well as binary displays where the colors of both disks mismatched the colors mentioned (i.e., situations incompatible with both conjunction and disjunction simulations).

#### Design and procedure

The experiment followed a 2 (Coordination: AND vs. OR) x 2 (Visual Stimuli: Two-Gestalts vs. One-Gestalt) factorial design. For filler trials, the experiment only contrasted the two connectives in the ‘Coordination’ condition, hence trials included only displays where stimuli had the same shape. A previous pilot study, where we used a fully factorial design, proved to be excessively difficult (i.e., yielded low accuracy scores). As in Experiment 1 and in Experiment 2, participants were informed as to what the correct responses in filler trials should be, but received no further details about the correct answer in the conditions of interest where stimuli colors matched both colors mentioned in conjunction and disjunction descriptions.

The structure of a trial was as follows. First, the word “Ready” appeared in the centre of the screen for 800 ms, followed by a 500 ms blank screen and by a visual display that remained visible until response. The experiment consisted of 6 practice trials and 72 experimental trials (24 target trials and 48 fillers) presented in individually randomized orders in two equal blocks. The dependent variables were the proportion of ‘yes’ responses and the response times across trials.

### Results and discussion

Fig 2F summarizes the validation patterns (i.e., average proportion of ‘yes’ responses) across trials. As in the previous experiments, we calculated accuracy scores for two logit models. The first model included the binomial dependent variable ‘response accuracy’ and ‘participants’ as random factors, as well as the predictor variables ‘coordination’ and ‘visual stimuli’ as fixed factors. We contrasted this model with a model that also included an interaction between the fixed factors. We found the second model to be a better fit for the data, χ2 = 35.12, p < .001. In particular, the log odds of validating disjunctions increased by 3.60 (Std. Error = 0.80, p < .001) compared to conjunctions when stimuli formed one Gestalt (i.e., when they were similar to each other). Filler trials scored were well above chance in the single condition tested (i.e., where stimuli had the same-shape). For conjunction first, second, and no-match trials, validation scores averaged .90, .94, and 1.00 respectively. For disjunction first, second, and no-match trials, validation scores averaged .84, .77, and .98 respectively.

Fig 2E summarizes the average response times across trials. We compared two models using LMER with and without an interaction between the fixed factors ‘coordination’ and ‘visual stimuli’. The interaction model was a better fit, χ2 = 15.92, p < .001. Participants responded on average 200 ms later (Std. Error = 49.95, t = 4.00) in one-Gestalt trials (where stimuli were similar to each other) than in two-Gestalts trials (where stimuli were different from each other) for the disjunction condition. These results confirm the accuracy data above, thus indicating a preference for associating conjunction simulations with displays containing similar stimuli and disjunction simulations with displays containing dissimilar stimuli.

## General Discussion

In three experimental studies, we provided support for the hypothesis that reasoning with conjunctions and disjunctions observes key Gestalt principles, which generate perceptual compatibility effects. In particular, high validation scores obtained for conjunctions describing perceptually-dependent stimuli and for disjunctions describing perceptually-independent stimuli, where dependency status was determined as follows. In Experiment 1, dependent and independent stimuli were moving simultaneously, respectively alternatively, thus illustrating the principle of common fate. In Experiment 2, dependent and independent stimuli were placed close to each other, respectively far from each other, thus illustrating the principle of proximity. In Experiment 3, dependent and independent stimuli exhibited similar shapes, respectively dissimilar shapes, thus illustrating the principle of similarity. The analysis of response times strongly supports these findings and further suggests that conjunction and disjunction meaning can override global-precedence effects, which otherwise apply systematically in visual perception. In particular, differences in accuracy scores and in response times between conditions were slightly greater in conjunction trials than in disjunction trials, as predicted by the global-precedence principle, which holds that global shapes (here, one-Gestalt displays) are processed faster than local shapes (here, two-Gestalts displays). However, participants were often faster to process two-Gestalts displays and slower to process one-Gestalt displays in disjunction trials compared to conjunction trials. It is thus remarkable that response times in disjunction trials indicate a weakening of the global-precedence effect in Experiment 1 and even a slight reversal in Experiment 2 and in Experiment 3.

We may surmise that conjunction simulations are easy to build because individuals are often willing to accommodate conjunction descriptions irrespective of stimuli perceptual properties. Indeed, the end result in conjunction contexts (i.e., both items mentioned are obtained) is the same irrespective of the practical circumstances where they occur. For example, when people consume soup and souffleé in sequence, they are still willing to describe them using a conjunction expression as long as they can perceive the two dishes as part of the same Gestalt (e.g., meal). In contrast, individuals are less willing to accommodate disjunction descriptions in imperfect contexts because dependency status impacts the outcome of the selection process. For example, individuals cannot use a disjunction expression to describe two dishes consumed in sequence unless they are able to represent the dishes as distinct Gestalts (i.e., by having the first dish for lunch and the second for dinner).

A comparison of effect sizes in validation scores across our experiments reveals that Gestalt grouping was strongest for stimuli varying in motion type and that it was weaker for stimuli varying in spatial placement and for stimuli varying in shape, thereby confirming previous findings reported in the literature on the relationship between grouping principles (e.g., [56], [62–64]) and suggesting that the effects induced by perceptual stimuli fall out from the principles governing the visual system.

Alternatively, asymmetries between visual cues can be attributed to a process of conceptual-metaphor formation [6–7]. According to these theories, people understand abstract concepts by automatically activating “image schemas”, which ground abstractions in experientially more basic concepts. Image schemas are analogue representations of spatial relations that may include concepts such as “source-path-goal”, “containment”, or “balance”. For example, Boot and Pecher [65] and Casasanto [66] showed that people understand concepts relating to stimuli shape (e.g., similarity) in terms of concepts relating to stimuli placement (e.g., closeness) but not the other way around. For example, in the study by Boot and Pecher, participants were faster to judge on whether two squares were similar in color when they were close than when they were far from each other. Also, they were faster to judge on whether two squares were dissimilar in color when they were far than when they were close to each other. Since the reverse was not true that is, color similarity did not influence distance decisions, the authors concluded that mappings between “similarity and “closeness” are asymmetrical because proximity is the more basic concept.

While the conceptual metaphor-formation theory might appear particularly compelling, at least at first sight, our results call into question two of its most important assumptions. The first assumption is the idea that people represent abstract concepts in terms of image schemas, suggesting that people can only manipulate a limited number of concepts from which they derive a large number of concepts. The second assumption is the idea that concepts must be situated at various levels of abstractness such that, for example, “shape” is a more abstract concept than “proximity”, which in turn is a more abstract concept than “motion type”. Yet the results of our studies suggest that any of the following scenarios is a plausible alternative. In the first scenario, understanding an abstract concept (e.g., “similarity”) would require instant access to the most concrete concept (e.g., “joint motion”). In the second scenario, understanding an abstract concept would require instant access to a more concrete concept (e.g., “closeness”). Hence we cannot confirm the existence of a hierarchical, level-based activation mechanism for abstract concept comprehension. Our results also fail to confirm the idea that an abstract concept must be understood in terms of a less abstract concept via the least abstract concept. In particular, our results fail to suggest that joint motion, proximity, and similarity afford a particular abstract representation. Whether and how image schemas combine into concept types is an outstanding issue that conceptual-metaphor theorists need to address.

We settled the issue of representation formation within the Gestalt-theoretical framework by providing evidence for independent effects of motion type, proximity, and similarity. In particular, we determined that any of these dimensions is a cue to visual dependency hence each of them can define the number of Gestalts involved in conjunction and in disjunction representations. More importantly, because differences in strength between grouping principles (i.e., common fate, proximity, and similarity) follow the rules of the human visual system, reasoners may be unaware of the differences and commonalities between these principles or indeed of the fact that conjunction and disjunction representations are Gestalt-like. Evidence from optical illusions supports the idea that people are unaware of the workings of the visual system. In particular, humans may unduly perceive contrasts between stimuli properties in certain contexts despite being unable to objectively measure them. Subsequent studies will shed more light on Gestalt awareness and on further characteristics of conjunction and disjunction simulations beyond the number of their constitutive elements. Part of our investigation will be to determine whether rich Gestalt simulations are instantly triggered as people process coordination descriptions. If so, we should be able to elicit perceptual compatibility effects not only in reasoning tasks but also in indirect tasks, where individuals are not asked to judge on the validity of conjunction and disjunction descriptions.
